# Immunonutrition vs. Standard Oral Nutritional Supplements in Prehabilitation for Colorectal Cancer Surgery: A Randomized Clinical Trial Assessing Morbidity and Body Composition by Computed Tomography

**DOI:** 10.3390/nu18203381

**Published:** 2026-10-15

**Authors:** Beatriz Ugalde, Luz D. Juez, Isabel Ruiz-Torres, Inés Perea-Fuentes, Sara Martos, Irene DeVicente, Daniel Melero, Javier Blázquez, José I. Botella-Carretero

**Affiliations:** 1 Department of Endocrinology and Nutrition, Hospital Universitario Ramón y Cajal, 28034 Madrid, Spain; beatriz.ugalde@salud.madrid.org; 2 Instituto Ramón y Cajal de Investigación Sanitaria (IRyCIS), 28034 Madrid, Spain; luzdivina.juez@salud.madrid.org (L.D.J.); dmelero1803@gmail.com (D.M.); javier.blazquez@salud.madrid.org (J.B.); 3 Department of General and Digestive Surgery, Hospital Universitario Ramón y Cajal, 28034 Madrid, Spain; ines.perea@salud.madrid.org (I.P.-F.); saramartos.mel@gmail.com (S.M.); irenedevicente@gmail.com (I.D.); 4 Department of Medicine, Universidad de Alcalá (UAH), 28801 Alcalá de Henares, Spain; 5 Department of Anesthesiology, Hospital Universitario Ramón y Cajal, 28034 Madrid, Spain; irtorres@salud.madrid.org; 6 Department of Radiology, Hospital Universitario Ramón y Cajal, 28034 Madrid, Spain

**Keywords:** immunonutrition, oral nutritional supplements, colorectal cancer, body composition, muscle mass, sarcopenia

## Abstract

**Background/Objectives**: Perioperative immunonutrition (IMN) has been proposed to improve outcomes after colorectal surgery. However, the evidence is heterogeneous, and few studies have assessed morbidity together with computed tomography (CT)-derived body composition. We compared IMN with standard oral nutritional supplementation (SONS) in patients undergoing colorectal cancer surgery, evaluating postoperative complications and changes in body composition. **Methods**: Two-arm open-label randomized clinical trial including sixty patients (SONS: n = 30; IMN: n = 30). Postoperative complications were assessed using the Clavien–Dindo classification, and the Comprehensive Complication Index (CCI). Body composition was quantified using pre- and postoperative CT scans, analyzing the skeletal muscle mass index (SMMI) and psoas area. **Results**: Baseline characteristics were comparable between groups, except for a higher age in the IMN group (*p* = 0.036). Overall morbidity was 40% in SONS and 50% in IMN (95%CI: −0.35 to 0.15). No differences were observed in the CCI (14.8 ± 23.1 vs. 15.7 ± 19.0, 95%CI: −11.8 to 10.0). Very major morbidity (Clavien ≥ IIIb) was comparable between groups (*p* = 0.687). At follow-up there was a decline in handgrip strength in SONS whereas it remained stable in IMN (*p* = 0.030 for the interaction). Also, there was a decrease in SMMI (−2.33, 95%CI: −1.19 to −3.48 cm^2^/m^2^) and psoas area (−1.15, 95%CI: −0.67 to −1.65 cm^2^) in SONS, whereas in IMN they remained stable: SMMI (−1.48, 95%CI: 1.04 to −3.99 cm^2^/m^2^), psoas area (−0.71, 95%CI: 0.36 to 1.78 cm^2^). **Conclusions**: IMN did not reduce morbidity compared with SONS when used as prehabilitation for colorectal cancer surgery. Exploratory findings suggested a possible attenuation of postoperative muscle loss with IMN.

## 1. Introduction

Colorectal cancer remains one of the most prevalent and lethal malignancies worldwide and in our country, imposing a substantial burden on global healthcare systems [1]. Despite significant advancements in oncological care, surgical resection continues to be the cornerstone of curative treatment [2]. However, major colorectal surgery is inherently associated with profound physiological stress, high rates of postoperative complications, and an accelerated state of muscle catabolism. These adverse outcomes not only prolong hospital stays and increase healthcare costs but can also compromise long-term oncological survival and delay adjuvant therapies [3].

The group that started the Enhanced Recovery After Surgery (ERAS) Society introduced their first consensus guideline in 2005 to improve the care of patients undergoing major colorectal surgery. When properly implemented, care pathways based on these guidelines have been shown to reduce morbidity rates, improve recovery, shorten lengths of stay, and reduce costs after major colorectal surgery [4]. Traditionally, postoperative management relied on reactive recovery; however, the clinical paradigm has shifted toward proactive optimization via prehabilitation—a multidisciplinary strategy aimed at enhancing a patient’s functional capacity prior to surgical stress, in which nutritional intervention constitutes a cornerstone [5,6].

In this regard, several studies have yielded contradictory results regarding the effects of immunonutrition (IMN) on postoperative outcomes for colorectal cancer. A multicenter randomized clinical trial demonstrated a decrease of more than half in the total number of complications with IMN compared with a control group, primarily due to a significant decrease in infectious complications [7]. Similarly, other randomized clinical trials showed a more than 10% decrease in wound infections [8], reduced hospital costs [9], and improvements in certain anthropometric measurements and biochemical parameters [10]. Conversely, other studies have failed to demonstrate these clinical benefits [11,12].

Consequently, in its most recent guidelines for patients undergoing colorectal cancer surgery, the ERAS Society recommends: (a) routine nutritional screening for all surgical candidates; (b) nutritional and protein supplementation (>1.2 g/kg/day) for at least 7 days for patients who are malnourished or at risk of malnutrition; and (c) the consideration of preoperative oral IMN for 5 to 7 days, despite moderate evidence and a weak recommendation strength [5].

Skeletal muscle mass has emerged as a critical and independent determinant of postoperative outcomes in patients with colorectal cancer. Sarcopenia, characterized by the progressive depletion of muscle mass and function, is highly prevalent in this oncological population due to tumor-induced systemic inflammation, cancer cachexia, and baseline malnutrition [13]. Accumulating clinical evidence demonstrates that low skeletal muscle mass and compromised muscular strength are strongly associated with an increased risk of severe surgical complications [14]. Whether IMN can effectively counteract postoperative muscle catabolism, maintain handgrip strength, and stabilize muscle mass during critical recovery phases remains to be fully elucidated. Therefore, the aim of our study was to analyze the effect of IMN versus standard oral nutritional supplements (SONS) on postsurgical complications together with changes in muscle mass and muscle strength.

## 2. Materials and Methods

### 2.1. Patients and Study Design

A two-arm, open-label, randomized clinical trial was conducted from 1 January 2024 to 31 December 2025 (https://clinicaltrials.gov—NCT07653737, 15 June 2026). The study was approved by the University Hospital Ramón y Cajal Ethics Committee (PI−20210406 on 9 May 2023 with the registration number 118/23). Sixty patients fulfilling the inclusion and exclusion criteria were enrolled after screening. All participants provided written informed consent. Inclusion criteria included being over 18 years of age, undergoing elective colorectal cancer surgery, and providing fully informed consent to participate. Exclusion criteria were as follows: emergency surgery, prior use of nutritional supplements, hypersensitivity to arginine or omega−3 fatty acids, inability to tolerate oral nutrition, and clinical conditions such as human immunodeficiency virus infection, pregnancy, uncontrolled infection, dementia or advanced degenerative neurological disease.

Study participants were evaluated during pre-anesthesia consultations through anthropometric measurements, a complete blood analysis, an American Society Anesthesiologists (ASA) evaluation and nutritional screening tests: the Controlling Nutritional Status (CONUT) score, the Malnutrition Universal Screening Test (MUST), and the Global Leadership Initiative on Malnutrition (GLIM) criteria. Hand dynamometry was performed using a Jamar dynamometer (Asimow Engineering Co., Los Angeles, CA, USA), and the mean of three measurements obtained from the dominant hand was recorded and expressed as percentiles for the age and sex of our reference population [15]. The same comprehensive assessment was repeated at the 90-day follow-up. Preoperative and 3-month follow-up computed tomography (CT) scans were used to extract data for the analysis of body composition.

### 2.2. Randomization and Intervention

Randomization was performed using opaque envelopes to assign patients to the two intervention groups. Sixty patients were randomized prior to surgery between January 2024 and December 2025. Patients were allocated to receive two 200 mL bricks per day of either an oral nutritional supplement enriched with arginine, nucleotides, vitamin D, and omega−3 fatty acids (Atempero®, Nutrisens Spain, Spain) for the IMN group, or a standard oral nutritional supplement (Tdiet HPR®, Nutrisens Spain, Spain) for the SONS group. The detailed composition of the products is available in Appendix A (Table A1).

The allocation to the intervention took place at least one week before the scheduled surgery, but because the final date of surgery could change owing to operating room scheduling requirements, the number of days of supplement intake varied among participants. Patients were instructed to record the amount of oral nutritional supplements consumed each day and to return their diaries at the end of the follow-up.

The investigator who generated the randomization sequence (J.I.B.-C.) was different from those who recruited the patients, and both the investigator who performed the anthropometric and nutritional assessments (D.M.) and the radiologist who analyzed body composition on CT (J.B.) were blinded to the intervention allocation.

Four patients were excluded: one due to the absence of definitive oncological pathology on the postoperative biopsy, and three due to being lost to follow-up, which prevented postsurgical re-evaluation. An intention-to-treat analysis was performed for all randomized patients, and a per-protocol analysis was performed on the 56 patients who completed the follow-up and had full data available, including body composition by CT.

### 2.3. Outcomes

The primary outcome of the study was the effect of IMN versus SONS on postsurgical complications. For this purpose, complication severity was assessed using the Clavien–Dindo classification [16], and the global burden was evaluated using the Comprehensive Complication Index (CCI) [17].

Secondary exploratory outcomes included the effect of IMN versus SONS on body composition quantified using the preoperative and the 90-day follow-up postoperative CT scans and changes in weight, body mass index (BMI), calf circumference, and handgrip strength.

### 2.4. Body Composition by CT Scans

The thinnest available axial abdominal slices (between 0.625 and 5 mm) at the mid-L3 vertebra level were selected for analysis. An experienced radiologist (intra-class correlation coefficient of 0.97) performed semi-automatic 2D segmentation and conducted all radiological measurements twice from this section, using the average as the final data. Visceral adipose tissue (VAT) was identified using an attenuation range of −150 to −50 HU, while subcutaneous adipose tissue (SAT) was defined using a range of −190 to −30 HU. Muscle tissue was identified using an attenuation interval of −29 to +150 Hounsfield units (HU).

Sarcopenia was diagnosed based on the skeletal muscle mass index (SMMI), calculated as the sum of the cross-sectional muscle areas at the L3 level (cm^2^) and normalized to the square of the patient’s height (m^2^). Sarcopenia was defined using L3 CT-derived cut-offs based on Prado et al. [18], with the BMI-dependent modifications published by Martin et al. [19]: SMMI < 43 cm^2^/m^2^ for males with a BMI < 25 and <53 cm^2^/m^2^ for males with a BMI ≥ 25, and <41 cm^2^/m^2^ for females.

### 2.5. Statistical Analysis

Sample size analysis was performed using the online tool GRANMO 8.0 [20]. Accepting an alpha risk of 0.05 and a statistical power greater than 0.8 in a two-sided test, 30 subjects are required in each group to detect a 30% reduction in the postsurgical complication rate.

For the primary endpoint, an intention-to-treat analysis was performed including all randomized patients, followed by a sensitivity analysis restricted to those who completed the follow-up (per-protocol analysis). Secondary outcomes were analyzed including only patients who completed the follow-up, as they had available postoperative body composition data. These results were therefore considered exploratory.

The Kolmogorov–Smirnov test was applied to continuous variables to assess normality. Logarithmic or square-root transformations were applied as needed to ensure a normal distribution. Results are expressed as mean ± standard deviation (SD) unless otherwise stated. Baseline comparisons between groups were performed using the independent Student’s t-test or the Mann–Whitney U test for continuous variables, and the χ^2^ test for categorical variables, as appropriate.

A repeated-measures general linear model (GLM) was employed to compare continuous variables throughout the study, defining the time points as the within-subject factor and the intervention group as the between-subject factor. Sphericity was assessed using Mauchly’s test. Binary logistic regression was performed to adjust the results of the primary outcome for age, using a backward conditional selection strategy. Analyses were performed using SPSS version 25 (SPSS Inc., Chicago, IL, USA). A *p*-value < 0.05 was considered statistically significant.

### 2.6. Use of Generative Artificial Intelligence

Generative artificial intelligence (GenAI) was used in this paper to generate graphics from original data of the study (ChatGPT; GPT-4o version, OpenAI Inc.; San Francisco, CA, USA and Claude; Claude 3.5 Sonnet version, Anthropic Inc.; San Francisco, CA, USA) and for translation and superficial text editing such as grammar, spelling and punctuation (Google Gemini; Gemini 1.5 Pro version, Google Inc.; Mountain View, CA, USA). GenAI-generated figures were subsequently verified from the authors’ original data to ensure its accuracy.

## 3. Results

**Figure 1:**
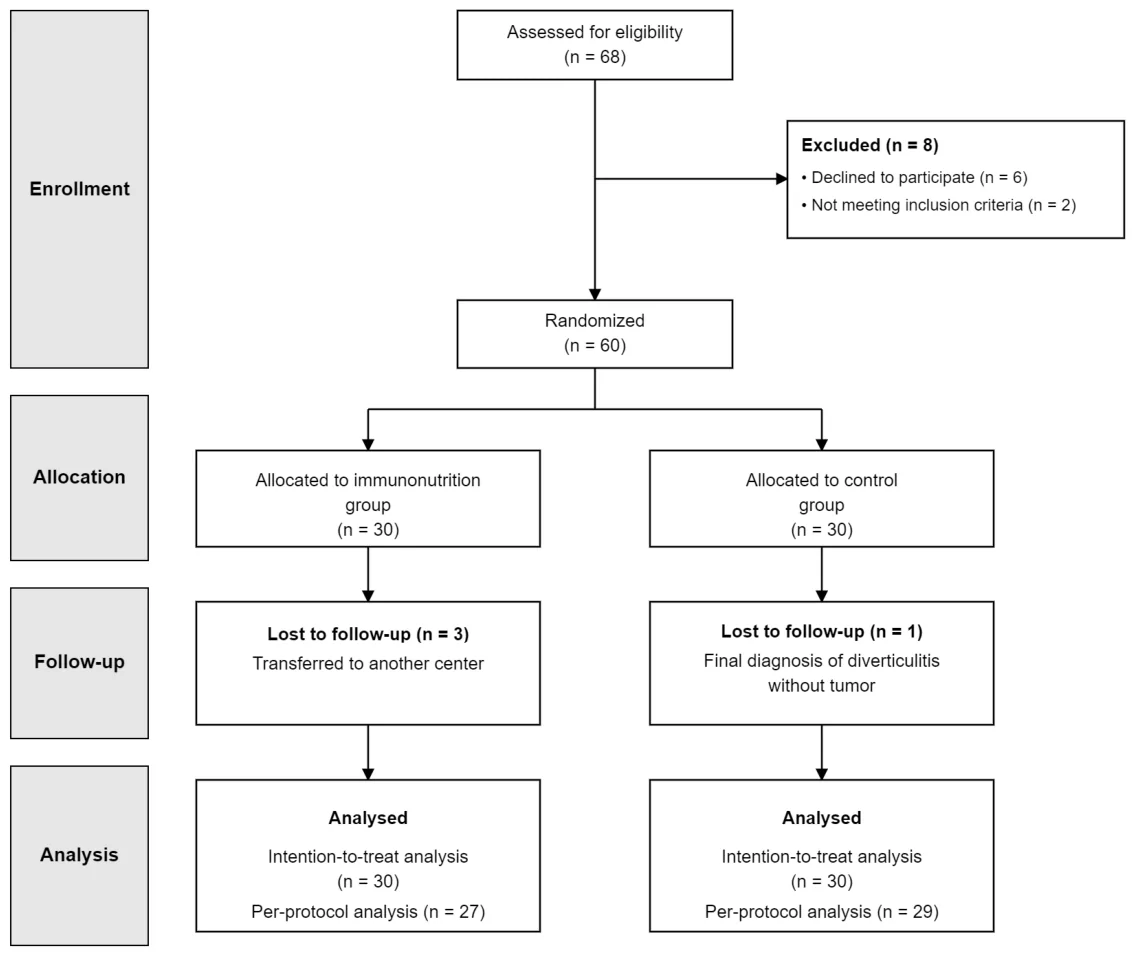
Flow chart of the study.

Sixty-eight patients were assessed for eligibility. Six refused to participate and two failed to meet the inclusion and exclusion criteria. Of the 60 randomized patients, four were excluded: one due to the absence of definitive oncological pathology on the postoperative biopsy (diverticulitis without evidence of tumor), and three due to loss to follow-up (referred to another center), which prevented postsurgical re-evaluation. An intention-to-treat analysis was performed for all randomized patients, and also a per-protocol analysis on the 56 patients who completed the follow-up and had full data available, including body composition assessed by CT (Figure 1).

All baseline clinical, biochemical and nutritional characteristics were comparable between groups, except for a higher mean age in the IMN group (*p* = 0.036) (Table 1).

### 3.1. Primary Outcomes

**Figure 2:**
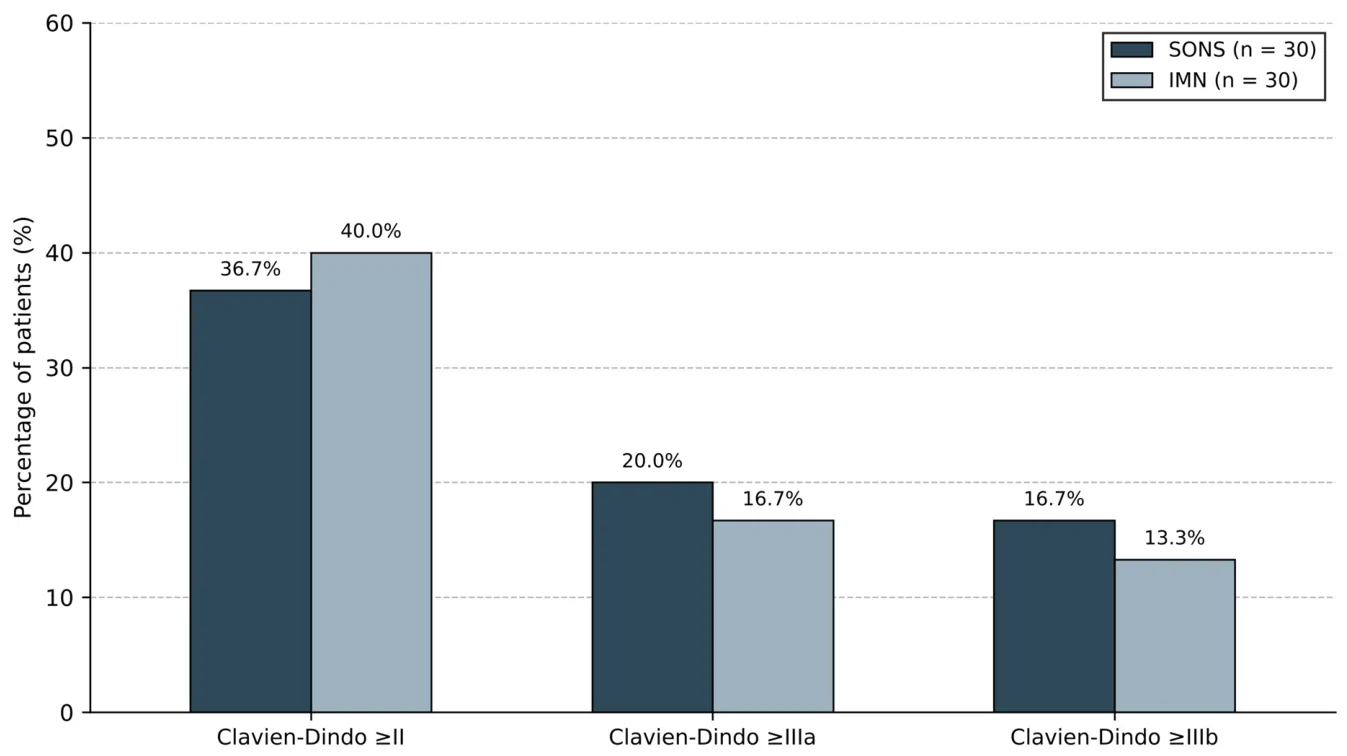
Postsurgical complications severity in both groups of interventions by the Clavien–Dindo classification in an intention-to-treat analysis. There were no significant differences (*p* > 0.05). SONS: standard oral nutritional supplements; IMN: immunonutrition.

In the intention-to-treat analysis, no significant differences were found in postoperative morbidity, complication rates, or overall complication burden as measured by the CCI (Table 2). Major morbidity (Clavien–Dindo grade ≥ IIIb) was also comparable between groups (Table 2 and Figure 2). The per-protocol sensitivity analysis showed similar results, with an overall morbidity of 41.4% in the SONS group and 51.9% in the IMN group (*p* = 0.432), a mean CCI of 15.3 ± 23.3 in the SONS group and 16.1 ± 19.1 in the IMN group (*p* = 0.891), and Clavien–Dindo grade ≥ IIIb complications in five and four patients respectively.

### 3.2. Secondary Outcomes

Because these outcomes were analyzed only in patients who completed follow-up and had postoperative body composition data available, they should be considered exploratory.

Weight loss occurred in both groups (−1.4 ± 0.2 Kg for SONS and −0.9 ± 0.4 Kg for IMN, *p* < 0.001 for the within factor effect) but with no difference between the interventions (*p* = 0.399 for the interaction). The same pattern was observed with calf circumference which showed a decline at follow-up (−0.7 ± 0.2 cm for SONS and −0.7 ± 0.1 cm for IMN, *p* < 0.001) with no difference between groups (*p* = 0.750).

Handgrip strength was similar at baseline between groups (P44.5 ± 25.9 for SONS vs. P44.8 ± 31.9 for IMN, *p* = 0.966). At follow-up there was a decline in handgrip strength in the SONS group (P39.3 ± 24.8, difference from baseline −P5.2, 95%CI: −P8.1 to −P2.3) whereas it remained stable in the IMN group (P45.5 ± 27.1, difference from baseline P0.7, 95%CI −P3.7 to P5.1; *p* = 0.030 for the interaction).

When analyzing changes in body composition by CT, there were no intergroup differences between both groups of intervention in preoperative body composition parameters (Table 3). Preoperatively, 60.7% of all patients presented with sarcopenia, 58.6% in the SONS group and 63.0% in the IMN group (*p* = 0.782). This classification was merely used descriptively to analyze the body composition changes as an exploratory analysis due to the lack of adequate statistical power.

**Figure 3:**
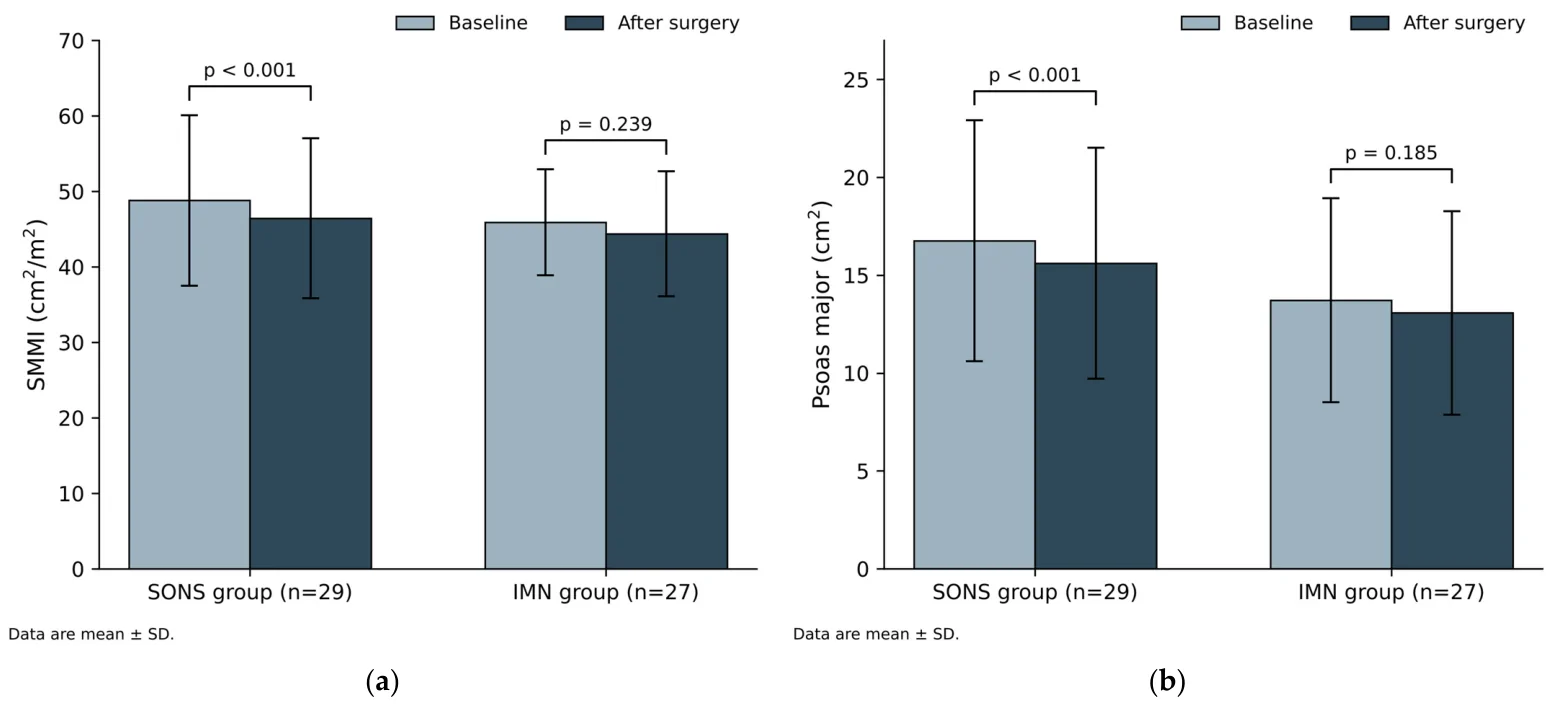
Changes in body composition by CT in all participants separated by intervention: (**a**) SMMI: skeletal muscle mass index; (**b**) psoas index. Data are mean ± SD. *p*-values correspond to within-group preoperative vs. postoperative comparisons.

**Figure 4:**
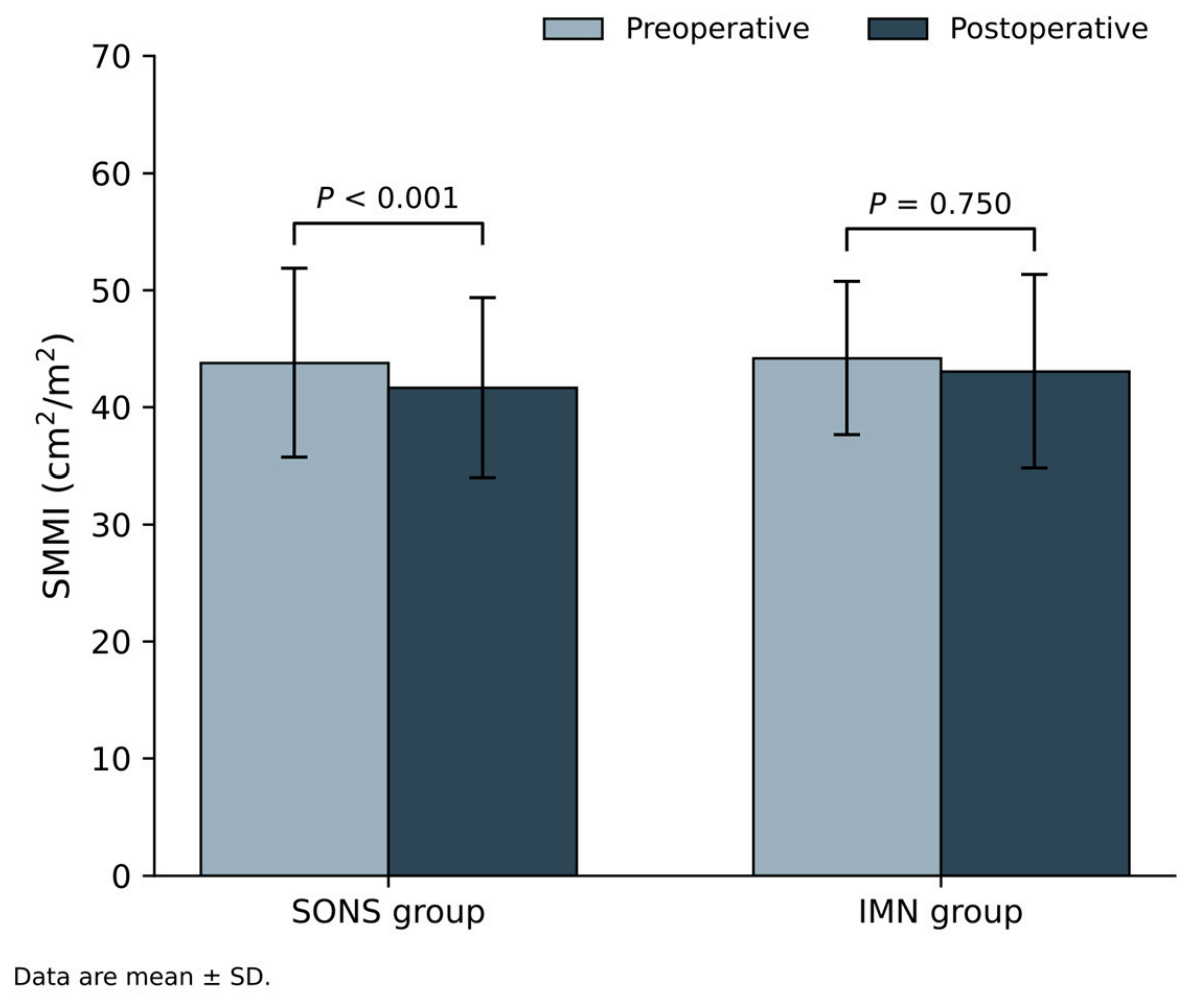
Changes in body composition by CT in those participants presenting with preoperative sarcopenia. Data are mean ± SD. *p*-values correspond to within-group preoperative vs. postoperative comparisons.

At follow-up, in the SONS group there was a decrease in SMMI (−2.33, 95%CI: −1.19 to −3.48 cm^2^/m^2^) and psoas area (−1.15, 95%CI: −0.67 to −1.65 cm^2^) in all patients (Table 3 and Figure 3), and in those with preoperative sarcopenia (Figure 4). Conversely, in the IMN group, SMMI (−1.48, 95%CI: 1.04 to −3.99 cm^2^/m^2^) and psoas area (−0.71, 95%CI: 0.36 to 1.78 cm^2^) were stable (Table 3 and Figure 3). Interestingly, both groups showed a decrease in visceral fat. However, the group by time interaction was not significant for any of the variables studied (Table 3), so these findings do not demonstrate a differential effect of IMN and they may be only hypothesis-generating results.

### 3.3. Ancillary Analyses

As age was different between both groups of intervention, binary logistic regression was performed to adjust the results of the primary outcome. For overall complications neither the intervention (*p* = 0.598) or age (*p* = 0.454) were significant (χ^2^ = −0.566, *p* = 0.452, R^2^ = 0.000). The same occurred for very major morbidity (Clavien ≥IIIb), with no effects of the intervention (*p* = 0.688) or age (*p* = 0.976) in the final model (χ^2^ = −0.162, *p* = 0.687, R^2^ = 0.000).

For secondary outcomes, in the repeated-measures analysis of handgrip strength, when age was introduced as a covariate, the result reported above remained significant (*p* = 0.040 for the interaction). Changes in body weight and calf circumference remained non-significant after age adjustment (interaction *p* = 0.357 and *p* = 0.640, respectively). For CT-derived body composition parameters, adjustment for age did not change the non-significant group by time interaction either.

### 3.4. Adherence and Adverse Events

Participants showed good adherence to the prescribed oral nutritional supplements, with a total of 9.9 ± 3.6 days before surgery for the SONS group and 11.2 ± 4.6 days for the IMN group (*p* = 0.238). Total reported ingested units did not differ between groups (18.1 ± 8.8 for SONS vs. 21.3 ± 10.4 for IMN, *p* = 0.214), nor did the number of units per day (1.8 ± 0.3 for SONS vs. 1.8 ± 0.4 for IMN, *p* = 0.270). There were no reported adverse events associated with either intervention.

## 4. Discussion

In this randomized clinical trial we demonstrated that prehabilitation with IMN prior to colorectal cancer surgery did not reduce overall morbidity, the severity of postsurgical complications, or the CCI compared to SONS. However, IMN showed a potential attenuation of postoperative muscle catabolism because at follow-up, although a significant decline in handgrip strength and SMMI was observed with SONS, both parameters remained stable in the IMN group.

In recent years, the clinical impact of IMN in colorectal surgery has been extensively evaluated across three pivotal meta-analyses. Khan et al. [21] analyzed 37 studies encompassing 3793 patients, reporting a significant reduction in overall postoperative infectious complications (Odds ratio OR 0.58, 95%CI 0.47–0.72) associated with IMN. Interestingly, subgroup analyses revealed that this benefit was strictly dependent on preoperative or perioperative administration, whereas postoperative initiation yielded no significant difference. Furthermore, this intervention significantly reduced surgical site infections (OR 0.65) and anastomotic leaks (OR 0.67), and shortened the length of hospital stay by 1.94 days, independent of the patients’ baseline nutritional status [21].

In agreement with these findings, Zhang et al. [22] evaluated 16 studies focused on colorectal cancer patients, demonstrating that preoperative IMN significantly lowered both postoperative infection rates (RR 0.56, *p* = 0.01) and wound infections (RR 0.44, *p* < 0.001). Although this specific meta-analysis did not find a significant reduction in length of hospital stay, it provided valuable mechanistic insights by demonstrating a significant increase in tumor-infiltrating lymphocytes—specifically CD16+ and CD56+ cells—in the tumor tissues of the IMN group, suggesting a heightened local immune response [22].

Conversely, the meta-analysis by McKechnie et al. [12], which compared preoperative IMN against conventional nutrition in 3337 patients, presented more conservative results. The authors reported a 30% relative risk reduction for surgical site infections (RR 0.70, *p* = 0.13) and a 44% reduction for anastomotic leaks (RR 0.56, *p* = 0.09); however, neither trend achieved statistical significance. Nonetheless, preoperative IMN did achieve a statistically significant decrease in postoperative hospital stay by 0.48 days. It is important to note, however, that the authors graded the overall certainty of evidence for all outcomes as low or very low [12].

Therefore, the pooled evidence from these recent meta-analyses indicates that preoperative and perioperative IMN generally optimizes clinical outcomes in colorectal surgery by mitigating infection rates and accelerating hospital discharge. However, discrepancies in statistical significance across studies and the low certainty of the current evidence emphasize the necessity for further robust, high-quality randomized controlled trials to establish definitive clinical guidelines [12,21,22].

Although our study failed to find benefits in postsurgical complications, we showed that IMN may potentially prevent the postsurgical decline in muscle mass and strength; however, these findings warrant confirmation in larger trials. As a novel aspect of our study, the evaluation of body composition emerges as a critical tool in oncology, shifting the focus from simple BMI to the qualitative assessment of skeletal muscle mass [23]. Among these parameters, sarcopenia has gained substantial recognition as a crucial independent prognostic factor in patients with colorectal cancer [14].

In the oncological context, sarcopenia is often driven by a complex interplay between tumor-mediated systemic inflammation, cancer cachexia, metabolic derangements, and patient-related factors such as malnutrition and physical inactivity [24]. Far from being a mere radiologic finding, sarcopenia represents a state of physiological vulnerability that deeply impairs the patient’s resilience against both the tumor and the physiological stress of therapeutic interventions [24]. The presence of sarcopenia exerts a profound negative impact on the survival outcomes of colorectal cancer patients. Accumulating evidence demonstrates a robust and consistent association between depletion of skeletal muscle mass and significantly compromised overall survival as well as disease-free survival [25]. Sarcopenic patients face a higher risk of systemic recurrence and cancer-related mortality compared to their non-sarcopenic counterparts. This survival disadvantage is mediated through multiple clinical pathways, and low muscle mass alters drug pharmacokinetics, leading to a higher incidence of dose-limiting toxicities during systemic chemotherapy [26,27].

Consequently, addressing sarcopenia is paramount to optimizing the therapeutic trajectory and survival of patients with colorectal cancer [28]. Given that muscle depletion can be accurately quantified on routine baseline staging CT scans using the SMMI at L3, it serves as an invaluable, readily available biomarker for risk stratification. Recognizing sarcopenia early provides a critical window of opportunity for multidisciplinary interventions [29]. Implementing personalized prehabilitation programs—combining targeted nutritional support, such as IMN, with tailored resistance and aerobic exercise—can stabilize or reverse muscle wasting [30].

The precise mechanisms by which IMN contributes to protecting muscle mass remain an area of ongoing investigation. However, the effects of dietary amino acids and IMN supplementation on cancer-induced skeletal muscle depletion have attracted significant interest in recent years [31]. For instance, an enteral diet enriched with IMN and whey-hydrolyzed peptides has been shown to exert anti-inflammatory effects and prevent cancer cachexia in the colon in 26 tumor-bearing mice treated with chemotherapy [32]. In clinical settings, a previous randomized, double-blind, multicenter trial demonstrated a significant increase in muscle mass via bioimpedance analysis exclusively in cancer patients receiving IMN, which is in close agreement with our findings [33].

Although outside the scope of our study, the dual anti-inflammatory and anabolic effect of IMN compared to SONS as a pathophysiological hypothesis was previously demonstrated in a prior study [34]. Patients receiving IMN exhibited a substantial reduction in systemic inflammatory mediators, specifically Tumor Necrosis Factor-Related Apoptosis-Inducing Ligand (TRAIL) and Lysosomal Associated Membrane Protein 3 (LAMP3). Simultaneously, there was an upregulation of galectin-1 (Gal-1), a pivotal protein involved in myogenic differentiation, muscle regeneration, and sarcolemma integrity, which enhances overall muscle function and tissue repair [35]. These findings highlight the potential of the IMN formula to activate intracellular signaling pathways essential for myogenesis. Taken together, these results demonstrate that the clinical benefits of IMN extend beyond mere caloric delivery, serving as an effective immunomodulatory intervention that simultaneously limits muscle catabolism and drives muscle regeneration in cancer patients [34].

Several limitations of our study need to be discussed. First, the relatively small sample size, which was initially powered to detect differences in postsurgical complications that ultimately did not manifest. Additionally, supplement intake was self-reported by patients rather than objectively measured, and physical exercise was not recorded, potentially introducing a confounding factor regarding changes in muscle mass [36]. Furthermore, the amounts of energy and protein provided by the two nutritional supplements differed; thus, any difference in muscle preservation cannot be attributed solely to IMN. Moreover, because dietary energy and protein intake was not assessed, the participants’ total intake could not be quantified, and its potential influence on the outcomes cannot be excluded.

## 5. Conclusions

Prehabilitation with IMN prior to colorectal cancer surgery did not reduce overall morbidity, the severity of postsurgical complications, or the CCI compared to SONS. Exploratory findings suggested a possible attenuation of postoperative muscle loss; however, these findings remain hypothesis-generating and must be confirmed in adequately powered studies.

## Figures and Tables

**Table 1:** Baseline characteristics of the included participants.

	SONS Group (N = 30)	IMN Group (N = 30)
Female sex	11 (36.7%)	10 (33.3%)
Age	68.1 ± 13.2	75.3 ± 12.8
ASA		
I	3 (10.0%)	3 (10.0%)
II	16 (53.3%)	16 (53.3%)
III	11 (36.7%)	10 (33.3%)
IV	0 (0.0%)	1 (3.3%)
Type 2 DM	4 (13.3%)	6 (20.0%)
Hypertension	9 (30.0%)	12 (40.0%)
Neo-adjuvant treatment	5 (16.6%)	6 (20.0%)
BMI (Kg/m^2^)	27.2 ± 3.7	26.5 ± 5.1
Calf circumference (cm)	35.5 ± 3.0	34.8 ± 4.0
C-reactive protein (mg/L)	11.5 ± 34.3	11.4 ± 17.9
Serum albumin (g/dL)	4.1 ± 0.6	4.0 ± 0.5
Serum prealbumin (mg/dL)	24.7 ± 7.0	22.3 ± 5.4
Total cholesterol (mg/dL)	176 ± 42	163 ± 31
Lymphocyte count (10^3^/µL)	2069 ± 757	1810 ± 691
CONUT		
0	16 (53.3%)	19 (63.3%)
1	8 (26.7%)	9 (30.0%)
2	6 (20.0%)	2 (6.7%)
MUST		
0	16 (53.3%)	17 (56.7%)
1	11 (36.7%)	10 (33.3%)
2	3 (10.0%)	3 (10.0%)
GLIM		
0	13 (43.3%)	13 (43.3%)
1	5 (16.7%)	6 (20.0%)
2	9 (30.0%)	9 (30.0%)
3	3 (10.0%)	2 (6.7%)
Tumor location		
Colon	27 (90.0%)	27 (90.0%)
Rectum	3 (10.0%)	3 (10.0%)
Minimal invasive surgery	25 (83.3%)	26 (86.7%)

Data are mean ± SD or n (%). SONS: standard oral nutritional supplements; IMN: immunonutrition; ASA: American Society Anesthesiologists evaluation; DM: diabetes mellitus; BMI: body mass index; CONUT: Controlling Nutritional Status score; MUST: Malnutrition Universal Screening Test; GLIM: Global Leadership Initiative on Malnutrition criteria.

**Table 2:** Postsurgical complication rates.

	SONS Group (n = 30)	IMN Group (n = 30)	Difference (95%CI) *
Total complications	12 (40.0%)	15 (50.0%)	−10.0 (−0.35 − 0.15)
Postsurgical complications	8 (26.7%)	13 (43.3%)	−16.7 (−38.1 − 7.2)
Clavien–Dindo			
I	1 (3.3%)	3 (10.0%)	−6.7 (−22.5 − 8.2)
II	5 (16.7%)	7 (23.3%)	−6.7 (−26.6 − 13.8)
IIIa	1 (3.3%)	1 (3.3%)	0.0 (−13.6 − 13.6)
IIIb	2 (6.7%)	2 (6.7%)	0.0 (−15.4 − 15.4)
IVa	2 (6.7%)	1 (3.3%)	3.3 (−10.8 − 18.2)
IVb	0 (0.0%)	1 (3.3%)	−3.3 (−16.7 − 8.3)
V	1 (3.3%)	0 (0.0%)	3.3 (−8.3 − 16.7)
Anastomotic leak	0 (0.0%)	3 (10.0%)	−10.0 (−25.6 − 3.1)
Postsurgical bleeding	3 (10.0%)	4 (13.3%)	−3.3 (−20.9 − 14.2)
Surgical wound infection	1 (3.3%)	2 (6.7%)	−3.3 (−18.2 − 10.8)
Intra-abdominal abscess	0 (0.0%)	2 (6.7%)	−6.7 (−21.3 − 5.7)
Paralytic ileus	3 (10.0%)	6 (20.0%)	−10.0 (−28.5 − 8.8)
Surgical evisceration	1 (3.3%)	2 (6.7%)	−3.3 (−18.2 − 10.8)
Total infectious complications	2 (6.7%)	5 (16.7%)	−10.0 (−27.6 − 7.4)
Reoperation	2 (6.7%)	4 (13.3%)	−6.7 (−23.7 − 10.0)
Total medical complications	9 (30.0%)	7 (23.3%)	6.7 (−15.4 − 27.9)
Respiratory failure	3 (10.0%)	3 (10.0%)	0.0 (−16.9 − 16.9)
Renal failure	2 (6.7%)	2 (6.7%)	0.0 (−15.4 − 15.4)
Heart failure	1 (3.3%)	2 (6.7%)	−3.3 (−18.2 − 10.8)
Total parenteral nutrition	5 (16.7%)	5 (16.7%)	0.0 (−19.3 − 19.3)
Pneumonia	2 (6.7%)	3 (10.0%)	−3.3 (−19.7 − 12.7)
Blood transfusion	8 (26.7%)	5 (16.7%)	10.0 (−11.0 − 30.1)
CCI (total score)	14.8 ± 23.1	15.7 ± 19.0	−0.9 (−11.8 − 10.0)

Data are mean ± SD or n (%). SONS: standard oral nutritional supplements; IMN: immunonutrition; CI: confidence interval; CCI: Comprehensive Complication Index. * 95%CI of the difference of proportions or means between groups.

**Table 3:** Body composition by CT at baseline and at 3-month revaluation.

	Baseline	After Surgery	Time Effect *p*	Interaction *p* *
SONS Group (n = 29)				
Body circumference (cm)	97.66 ± 12.44	100.96 ± 11.05	0.794	
Subcutaneous fat (cm^2^)	140.40 ± 73.83	139.52 ± 59.51	0.899	
Visceral fat (cm^2^)	187.73 ± 111.6	182.95 ± 103.21	0.005	
SMMI (cm^2^/m^2^)	48.79 ± 11.27	46.46 ± 10.59	<0.001	
Psoas major (cm^2^)	16.76 ± 6.16	15.61 ± 5.90	<0.001	
IMN Group (n = 27)				
Body circumference (cm)	99.08 ± 13.54	99.16 ± 13.44	0.200	0.521
Subcutaneous fat (cm^2^)	138.10 ± 79.5	152.35 ± 87.25	0.473	0.569
Visceral fat (cm^2^)	213.79 ± 111.77	173.79 ± 115.21	0.001	0.707
SMMI (cm^2^/m^2^)	45.89 ± 7.01	44.41 ± 8.28	0.239	0.441
Psoas major (cm^2^)	13.72 ± 5.22	13.07 ± 5.21	0.185	0.355

Data are mean ± SD. * *p* for the interaction of group of intervention by time. SMMI: skeletal muscle mass index.

## Data Availability

Restrictions apply to the availability of the data generated or analyzed during this study to preserve patient confidentiality or because they were used under license. The corresponding author will, on request, detail the restrictions and any conditions under which access to some data may be provided.

## Abbreviations

The following abbreviations are used in this manuscript:

ASA	American Society Anesthesiologists
BMI	Body mass index
CCI	Comprehensive Complication Index
CI	Confidence interval
CONUT	Controlling Nutritional Status
CT	Computed tomography
DM	Diabetes mellitus
ERAS	Enhanced recovery after surgery
Gal-1	Galectin-1
GLIM	Global Leadership Initiative on Malnutrition
HU	Hounsfield units
IMN	Immunonutrition
LAMP3	Lysosomal Associated Membrane Protein 3
MUST	Malnutrition Universal Screening Test
OR	Odds ratio
SAT	Subcutaneous adipose tissue
SD	Standard deviation
SMMI	Skeletal muscle mass index
SONS	Standard oral nutritional supplements
TRAIL	Tumor Necrosis Factor-Related Apoptosis-Inducing Ligand
VAT	Visceral adipose tissue

## Appendix A

### Detailed Composition or the Oral Nutritional Supplements

Patients were allocated to receive at least one week before surgery either two bricks per day of oral nutritional supplements enriched with arginine, nucleotides, vitamin D and omega-3-fatty acid (Atempero^R^, Nutrisens Spain) for the group of IMN or two bricks per day of standard oral nutritional supplements (TdietHP^R^, Nutrisens Spain) for the group of SONS.

**Table A1:** Composition of the oral nutritional supplements per 100 mL.

	Atempero^R^	TdietHP^R^
Energy (kcal)	151	125
Proteins (g)	8.3	6.3
Arginine (g)	1.0	-
Carbohydrates (g)	17.3	13.1
Fat (g)	5.0	4.9
Omega-3 (g)	0.8	0.13
Vitamin D (µg)	3.6	3.0
Nucleotides (mg)	200	-
Fiber (g)	1.7	-
